# Depletion of *Kif13b* promotes neuroinflammatory attack against myelin in an experimental autoimmune encephalomyelitis mouse model

**DOI:** 10.1016/j.gendis.2025.101946

**Published:** 2025-11-19

**Authors:** Jingxuan Chen, Yitong Xu, Guolin Miao, Liwen Zheng, Kaikai Lu, Wenxi Zhang, Wei Huang, Yuhui Wang, Xunde Xian

**Affiliations:** a Institute of Cardiovascular Sciences, State Key Laboratory of Vascular Homeostasis and Remodeling, School of Basic Medical Sciences, Peking University, Beijing 100191, China; b Department of Cardiology and Institute of Vascular Medicine, Peking University Third Hospital, Beijing 100191, China; c Beijing Key Laboratory of Cardiovascular Receptors Research, Peking University Third Hospital, Beijing 100191, China

Multiple sclerosis (MS) is an inflammatory demyelinating neurodegenerative disease and the leading cause of disability in young people without any therapeutic treatment to date.^1^ The kinesin family is a molecular motor responsible for forward transport of mitochondria, RNA, proteins, and synaptic vesicles, mediating physiological and pathological processes, such as microtubule growth, axon development, and myelin degeneration.^2^ An abnormal number of kinesins during axonal transport may serve as biomarkers for neurodegenerative diseases. Recent studies have identified kinesin family member 13b (KIF13B), the largest family member, as an unconventional signal transduction regulator involved in myelin formation in the central and peripheral nervous systems.^3^ Nevertheless, whether KIF13B regulates the repair of myelin loss and its implication in the pathogenesis of MS has not been explored yet.

Based on the RNA-sequencing data provided by the GEO database (GSE224377) for patients with progressive MS, we found that the mRNA expression level of human *KIF13B* was significantly decreased with the most pronounced reduction among significantly altered kinesin family members in the region of chronic demyelinating lesions compared with the match normal-appearing white matter, suggesting that KIF13B may be closely related to the development of MS (Fig. 1A–C). Since MS samples are not readily available, animal models of experimental autoimmune encephalomyelitis (EAE) have been widely used to study the pathogenesis of MS, given that this murine model has very similar pathological changes to patients with MS, characterized by the presence of mononuclear cell infiltration around small blood vessels and demyelination in the central nervous system. For our study, we selected 8-week-old C57/B6J mice to establish a mouse model of EAE immunized with MOG35-55 and pertussis toxin. Complete Freund's adjuvant (CFA) and equal doses of pertussis toxin were used in control mice, and the lumbar spinal cords of the mice were harvested on day 21 (Fig. 1D). We then examined KIF13B mRNA and protein expression levels in the lumbar spinal cord of mice and observed a significant down-regulation of KIF13B expression in the lesion spinal cord of EAE compared with healthy controls (Fig. 1E, F). To clarify whether there is a causal effect between KIF13B and the onset of MS, we generated global *Kif13b* knockout (*Kif13b*^*−/−*^) mice and used wild-type (WT) mice from the same litter as controls, both of which were administered MOG35-55 and pertussis toxin to induce EAE. After 10 days of induction, we continuously monitored and clinically scored the behavior phenotypes. *Kif13b*^*−/−*^ mice had significantly higher behavioral scores and increased cumulative disease severity from day 17 after induction (Fig. 1G , H). As myelin loss is a key feature of EAE, we next investigated whether targeting KIF13B affected myelin loss in the spinal cord of EAE. Analysis based on Luxol fast blue staining results demonstrated that *Kif13b*^*−/−*^ mice had large areas of myelin loss and significantly higher demyelination scores (Fig. 1I, M). We further analyzed motor neuron damage in the anterior horn of the spinal cord by Nissl staining, showing that *Kif13b*^*−/−*^ mice had considerably reduced numbers of healthy neurons compared with WT mice at the same site in the lumbar spinal cord (Fig. 1J, M). To understand the cause of the exacerbation of myelin loss by *Kif13b* deletion, we focused on the attack of inflammatory cells against neuromyelin. Hematoxylin-eosin staining indicated that the area of motor neuron lesions in the anterior peduncle of the spinal cord had a higher concentration of mononuclear cells due to *Kif13b* deficiency and a marked elevation in neuroinflammatory scores (Fig. 1K, M). Immunofluorescence staining further confirmed that both astrocytes with GFAP-positive staining and microglia with IBA1-positive signal were greatly increased in the spinal cord of *Kif13b*^*−/−*^ mice, along with more severe neuronal damage (Fig. 1L, M). Thus, our data suggested a protective effect of KIF13B on neuroinflammation. Next, we sought to investigate the underlying mechanism by which KIF13B maintained inflammatory homeostasis using transcriptomic sequencing technology. The lumbar spinal cords of WT and *Kif13b*^*−/−*^ mice were harvested for RNA-sequencing analysis, and the differentially expressed genes were subjected to intersection analysis with MS-related genes in the NCBI database and screened for genes co-regulated in the context of *Kif13b* deletion and MS pathogenesis (Fig. 1N). We found that MER proto-oncogene tyrosine kinase (*Mertk)* is the top gene with the most significant change in our case (Fig. 1N). Previously, *Mertk*, a pro-efferocytosis gene highly expressed in microglia, has been reported to be a key gene associated with MS and is required for myelin regeneration in microglia.^4^ Consistently, our results from Western blotting and immunofluorescence staining revealed a significant decrease in MERTK protein level in the lumbar spinal cord of *Kif13b*^*−/−*^ mice relative to WT mice (Fig. 1O, P), suggesting that the protective effect of KIF13B on neuroinflammation and myelin regeneration may be attributable to normal MERTK expression. In agreement with the previous findings that KIF13B can stabilize MERTK in immune cells, such as macrophages,^5^ these new lines of evidence further support the concept that KIF13B is a critical regulator of MERTK. Therefore, it will be interesting to validate the therapeutic efficacy of KIF13B or MERTK overexpression for EAE in the context of KIF13B deficiency *in vivo* and *in vitro* experiments within glial cells. Importantly, screening loss-of-function mutations of *KIF13B* in large-scale cohorts of MS patients will be conducted in future studies for a better understanding of the precise role and the translational significance of KIF13B in MS progression. Furthermore, while the journey from basic research to clinical translation presents exciting opportunities, particularly regarding how to formulate KIF13B into a druggable complex and then deliver it across the blood–brain barrier, further investigation is warranted. Meanwhile, we will continue studying the functional domains of KIF13B in MS disease, aiming to enhance its clinical translational potential as a therapeutic target through strategies such as constructing targeted peptides or identifying small-molecule agonists.

**Figure 1:**
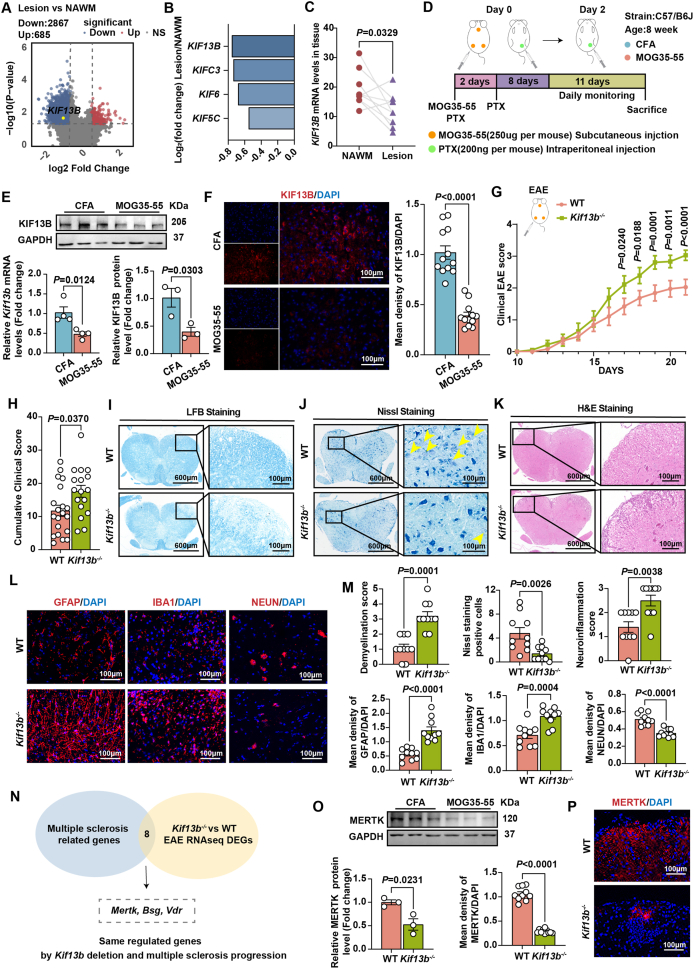
KIF13B is required for maintaining inflammatory homeostasis of the central nervous system and enhancing myelin regeneration in multiple sclerosis (MS). **(A)** The volcano plot displays differentially expressed genes (DEGs) between normal-appearing white matter (NAWM, *n* = 9) or lesion area (*n* = 9) from patients with MS in a GEO dataset (GSE224377). Up-regulated and down-regulated genes are highlighted in red and blue, respectively. Genes with a *P*-value < 0.05 and an absolute Log_2_fold change > 0.5 were defined as DEGs. **(B)** The bar graph displays kinesin family member genes with a *P*-value < 0.05 and an absolute fold change > 0.5 in GSE224377, ranked by Log_2_ fold change. **(C)** Analysis of human *KIF13B* mRNA expression in GSE224377. **(D–F)** EAE model mice and control mice were constructed with injections of MOG35-55 or CFA and pertussis toxin. (D) Schematic diagram of experimental design. **(E)** Quantitative PCR (*n* = 4/group) and Western blotting (*n* = 3/group) analysis of mouse KIF13B mRNA and protein expression in two groups. **(F)** Representative immunofluorescence staining images of KIF13B (red) protein levels (*n* = 12/group). **(G**–**P)** WT and *Kif13b*^*−/−*^ mice under EAE conditions. (G) Clinical EAE score of WT (*n* = 19) and Kif13b^*−/−*^ (*n* = 18) mice. (H) Cumulative clinical score. (I–M) Representative images of Luxol fast blue (LFB) staining, Nissl staining (yellow arrows indicate positive staining), hematoxylin-eosin (H&E) staining, and immunofluorescence staining of GFAP (red), IBA1 (red), and NEUN (red) protein levels and statistical analysis in the two genotypes (*n* = 10/group). (N) Combined analysis of co-regulated genes with *Kif13b* deletion and involved in MS pathogenesis. (O) Western blotting analysis of mouse KIF13B protein levels in two groups (*n* = 3/group). (P) Representative immunofluorescence staining images of MERTK (red) protein levels in two genotypes (*n* = 10/group). All experimental procedures were approved by Peking University Health Science Center (LA2010-059). All data were presented as mean ± standard error of the mean. The data in (C) were analyzed by the Wilcoxon matched-pairs signed-rank test, and the data in (E, F, M, O, P) were analyzed by the unpaired Student's *t*-test using Prism 9.

In conclusion, our study reports for the first time that motor protein KIF13B suppresses inflammatory infiltration, demyelination, and neuronal inactivation in the central nervous system by positively regulating the expression of the pro-efferocytosis molecule MERTK, providing a new potential therapeutic target for the interventional strategy of MS in future clinical trials.

## CRediT authorship contribution statement

**Jingxuan Chen:** Writing – original draft, Visualization, Methodology, Investigation, Formal analysis, Data curation, Conceptualization. **Yitong Xu:** Writing – original draft, Methodology. **Guolin Miao:** Methodology. **Liwen Zheng:** Methodology. **Kaikai Lu:** Methodology. **Wenxi Zhang:** Methodology. **Wei Huang:** Supervision, Formal analysis. **Yuhui Wang:** Supervision, Funding acquisition. **Xunde Xian:** Writing – review & editing, Validation, Supervision, Resources, Project administration, Methodology, Funding acquisition, Formal analysis, Conceptualization.

## Ethics declaration

Approval for all animal experiments was granted by the Laboratory Animal Ethics Committee of Peking University (LA2023460).

## Data availability

All data are available in the main text or the supplementary materials. The publicly available datasets analyzed to assess *Kif13b* transcription profiles during the current study are available in the Gene Expression Omnibus repository with the accession code GSE224377. The datasets generated and analyzed during the current study are available from the corresponding author upon reasonable request.

## Funding

This work was supported by the 10.13039/501100001809National Natural Science Foundation of China (No. 82270479, 82070460, HY2021-1), Beijing Natural Science Foundation (China) (No. 7242084 to Xunde Xian), Fundamental Research Funds for the Central Universities (China) (to Xunde Xian), Peking University Medicine plus X Pilot Program-Platform Construction Project (China) (No. 2024YXXLHPT010 to Xunde Xian), and the National Key Research and Development Program of China from the Ministry of Science and Technology (No. 2021YFF0702802 to Yuhui Wang).

## Conflict of interests

Xunde Xian is the member of Genes & Diseases Editorial Board. To minimize bias, he/she was excluded from all editorial decision-making related to the acceptance of this article for publication. The remaining authors declared no competing interests.
