# Supplementary material for: Depletion of Kif13b promotes neuroinflammatory attack against myelin in an experimental autoimmune encephalomyelitis mouse model
Source: Genes Dis. 2025 Nov 19;13(5):101946. doi: 10.1016/j.gendis.2025.101946 (PMC13101677; doi:10.1016/j.gendis.2025.101946)
Supplement: Multimedia component 1 [file mmc1.docx]

**Materials and Methods**

The data that support the findings of this study are available from the corresponding author on reasonable request.

**Animal Studies**

Our study examined male and female animals, and similar findings are reported for both sexes. All animal experiments were conducted in accordance with the National Institutes of Health Guide for the Care and Use of Laboratory Animals (NIH Publications, 8th edition, 2011) and approved by the Animal Ethics Committee of Peking University (LA2023460). Global Kif13b knockout (Kif13b^-/-^) and Kif13b flox (Kif13b^f/f^) mice were generated by the Institute of Laboratory Animal Science, Chinese Academy of Medical Sciences and Peking Union Medical College (Beijing, China) using CRISPR/Cas9 technology. All experimental animals were bred under pathogen-free conditions at the Animal Facility of Peking University Health Science Center.

**Scoring standard**

Clinical behavioural phenotype was scored as: 0, normal; 1, limp tail; 2, weak hindlimbs; 3, complete hindlimb paralysis; 4, complete hindlimb paralysis with some forelimb weakness; 5, moribund/dead.

The neuroinflammation was scored as: 0, no infiltration of inflammatory cells; 1, a few scattered inflammatory cells; 2, inflammatory cells infiltrate around blood vessels; 3, infiltrated inflammatory cells formed extensive perivascular cuffing extending to adjacent parenchyma, or inflammatory cells infiltrated into parenchymal without obvious cuffing.

Spinal cord demyelination was scored as: 1 = traces of subpial demyelination; 2 = marked subpial and perivascular demyelination; 3 = confluent perivascular or subpial demyelination; 4 = massive perivascular and subpial demyelination involving one half of the spinal cord with presence of cellular infiltrates in the CNS parenchyma; and 5 = extensive perivascular and subpial demyelination involving the whole cord section with presence of cellular infiltrates in the CNS parenchyma.

Counts of NISSL-positive cells were made in motor neuron regions of the anterior horn of the spinal cord of equal size, and the average of the contralateral counts was taken.

**Pathological analysis**

Pathological and analytical methods can be found in the previous work of the laboratory. For immunofluorescence staining, the stainings of KIF13B, GFAP, IBA1, NEUN and MERTK in spinal cords were analyzed using primary antibodies against KIF13B (1:200, SAB2101257, Sigma, Germany), GFAP antibody (1:200, PB9082, Bioss, CHINA), IBA1 (1:200, BSM-54132R, Bioss, CHINA), NEUN (1:200, BS-1613R, Bioss, CHINA)and MERTK antibody (1:200, ab300136, Abcam, UK), respectively.

**Statistical analysis**

All statistical analysis was performed using GraphPad Prism version 9.0 (GraphPad Software). Continuous data are presented as mean ± SEM. Normality of data distribution was assessed by the Shapiro-Wilk test and equality of variance by the Brown-Forsythe test. For comparisons between two groups, differences were compared using unpaired Student's t-test (normal distribution and equal variance) or Mann-Whitney U-test (non-normal distribution) or Welch t-test (unequal variances). For multiple comparisons of equal variance (>2 groups), one-way ANOVA analyses (single variable) or two-way ANOVA analyses (multiple variables) were used, followed by Tukey post hoc tests (equal variance). Kruskal-Wallis test and Bonferroni post hoc test were used for multiple tests of unequal variance. Values of P<0.05 were considered statistically significant.
